# GO-Y078, a Curcumin Analog, Induces Both Apoptotic Pathways in Human Osteosarcoma Cells via Activation of JNK and p38 Signaling

**DOI:** 10.3390/ph14060497

**Published:** 2021-05-24

**Authors:** Peace Wun-Ang Lu, Renn-Chia Lin, Jia-Sin Yang, Eric Wun-Hao Lu, Yi-Hsien Hsieh, Meng-Ying Tsai, Ko-Hsiu Lu, Shun-Fa Yang

**Affiliations:** 1 Morrison Academy Taichung, Taichung 406, Taiwan; lup@mca.org.tw; 2 Department of Orthopedics, Chung Shan Medical University Hospital, Taichung 402, Taiwan; cshy594@csh.org.tw; 3 School of Medicine, Chung Shan Medical University, Taichung 402, Taiwan; 4 Division of Hyperbaric Oxygen Therapy and Wound Medicine, Chung Shan Medical University Hospital, Taichung 402, Taiwan; 5 Institute of Medicine, Chung Shan Medical University, Taichung 402, Taiwan; gazn_sheep@yahoo.com.tw (J.-S.Y.); hyhsien@csmu.edu.tw (Y.-H.H.); vickyfatfat5252@gmail.com (M.-Y.T.); 6 Department of Medical Research, Chung Shan Medical University Hospital, Taichung 402, Taiwan; 7 American School in Taichung, Taichung 406, Taiwan; 21ericl@ast.tc.edu.tw

**Keywords:** GO-Y078, osteosarcoma, apoptosis, JNK

## Abstract

Osteosarcoma is the most common primary bone malignancy in teenagers and continues to confer a generally poor prognosis due to its highly metastatic potential. Poor solubility in water and instability of curcumin limits its bioavailability for use in the adjuvant situation to improve the prognosis and the long-term survival of patients with osteosarcoma. To further obtain information regarding the apoptosis induced by a new curcumin analog, GO-Y078, in human osteosarcoma cells, flow cytometric analysis, annexin V-FITC/PI apoptosis staining assay, human apoptosis array, and Western blotting were employed. GO-Y078 dose-dependently decreased viabilities of human osteosarcoma U2OS, MG-63, 143B, and Saos-2 cells and induced sub-G1 fraction arrest and apoptosis in U2OS and 143B cells. In addition to the effector caspase 3 and poly adenosine diphosphate-ribose polymerase, GO-Y078 significantly activated both initiators of extrinsic caspase 8 and intrinsic caspase 9, whereas cellular inhibitors of apoptosis 1 (cIAP-1) and X-chromosome-linked IAP (XIAP) in U2OS and 143B cells were significantly repressed. Moreover, GO-Y078 increased phosphorylation of extracellular signal-regulated protein kinases (ERK)1/2, c-Jun N-terminal kinases (JNK)1/2, and p38 in U2OS and 143B cells. Using inhibitors of JNK (JNK-in-8) and p38 (SB203580), GO-Y078′s increases in cleaved caspases 8, 9, and 3 could be expectedly suppressed, but they could not be affected by co-treatment with the ERK inhibitor (U0126). Altogether, GO-Y078 simultaneously induces both apoptotic pathways and cell arrest in U2OS and 143B cells through activating JNK and p38 signaling and repressing IAPs. These findings contribute to a better understanding of the mechanisms responsible for GO-Y078′s apoptotic effects on human osteosarcoma cells.

## 1. Introduction

Osteosarcoma is not only the most common primary bone cancer in children and adolescents but is also accountable for one of the most lethal pediatric malignancies [1,2,3]. The evaluation of patients with osteosarcoma should include plain radiographs and magnetic resonance imaging of the entire affected bone, which generally involves the metaphyses of the distal femur or proximal tibia [1]. As an early metastatic tumor, surgical en bloc resection or amputation of the extensively diseased extremity to achieve a complete radical excision still resulted in poor prognosis [1,4]. Chemotherapy is a cornerstone of treatment for osteosarcoma, so neoadjuvant chemotherapy followed by limb salvage surgery has increased long-term survival rates to approximately 68% recently [4,5]. While there are four pathologic subtypes of conventional high-grade osteosarcoma: osteoblastic, chondroblastic, fibroblastic, and telangiectatic, reflecting the predominant form of tumor matrix, no significant differences in outcome are associated with the various subtypes [1,2]. To date, potent metastatic lung transfers are still responsible for the unacceptable treatment failures and mortality rates [2,6]. Eventually, novel adjuvant agents that target osteosarcoma to cause cell death need to be developed and the precious molecular mechanisms of the cytotoxic activity remain to be elucidated.

Apoptosis is a form of programmed cell death that is characterized by typical morphological and biochemical hallmarks, including membrane blebbing, cell shrinkage, chromatin condensation, and nuclear DNA fragmentation [7]. To undergo apoptosis, activation of important initiator and effector caspases would be initiated through stimulation of the extrinsic (death receptor) pathway or the intrinsic (mitochondria) pathway [8,9,10]. All signaling eventually converges on the initiator caspases 8 or 9 and their downstream effector caspases 3 or 7. Moreover, multiple stress-inducible molecules, such as mitogen-activated protein kinase (MAPK)/extracellular signal-regulated protein kinase (ERK), c-Jun N-terminal kinase (JNK), and p38, have been implied in transmitting the apoptotic pathway [11,12]. On the other hand, both initiator and effector caspases are further regulated by the inhibitors of apoptosis (IAPs) family, which comprises a class of substantial apoptosis modulators that promote cell resistance to apoptosis, including cellular IAP 1 and 2 (cIAP-1 and 2) and X-chromosome-linked IAP (XIAP) [13].

While cIAPs modulate the expression of anti-apoptotic proteins to prevent the assembly of proapoptotic signaling complexes, XIAP is the most potent direct inhibitor of the apoptosis executioner, the caspases [13]. IAP overexpression in osteosarcoma contributes to cancer cell survival, chemo-resistance, disease progression, and poor prognosis, possibly rendering osteosarcoma resistant to standard chemotherapeutics and radiation therapy, so the obstacles make IAP proteins promising targets for therapeutic intervention [14,15,16]. For example, anti-cancer agents of doxorubicin and cisplatin sensitize osteosarcoma cells to tumor necrosis factor-related apoptosis-inducing ligands through downregulating IAP molecules [17]. Currently, most anti-cancer strategies in clinical oncology focus on triggering apoptosis in cancer cells, and failure to undergo apoptosis may result in treatment resistance. Thereby, understanding the molecular events that regulate apoptosis in response to chemotherapy provides novel opportunities to develop the targeted therapy through anti-apoptotic proteins and the intrinsic and/or extrinsic pathways for the metastatic osteosarcoma.

**Figure 1:**
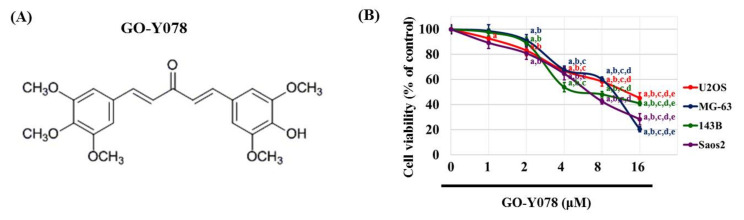
Effects of GO-Y078 on the cell viability of U2OS, MG-63, 143B, and Saos-2 cells. (**A**) The structure of curcumin analog GO-Y078. (**B**) The viability of U2OS, MG-63, 143B, and Saos-2 cells treated with GO-Y078 (1, 2, 4, 8, and 16 μM) for 24 h was detected by MTT assay and the effects are illustrated after quantitative analysis. Results are shown as mean ± S.D. ANOVA analysis with Scheffe’s posteriori comparison was used. U2OS (*n* ≥ 7): F = 314.386, *p* < 0.001; MG-63: F = 863.541, *p* < 0.001; 143B (*n* ≥ 4): F = 453.149, *p* < 0.001; Saos-2 (*n* ≥ 8): F = 451.896, *p* < 0.001. ^a^ Significantly different, *p* < 0.05, when compared to control. ^b^ Significantly different, *p* < 0.05, when compared to 1 μM. ^c^ Significantly different, *p* < 0.05, when compared to 2 μM. ^d^ Significantly different, *p* < 0.05, when compared to 4 μM. ^e^ Significantly different, *p* < 0.05, when compared to 8 μM.

Curcumin (diferuloylmethane), a bright yellow chemical produced by *Curcuma longa* plants, possesses diverse anti-cancer properties such as inhibition of tumor growth, induction of apoptosis, and suppression of metastasis through the modulation of multiple cell signaling pathways [18,19,20]. The vigorous cytotoxic activity of curcumin on osteosarcoma cells has been reported to be mediated by the induction of multiple apoptotic processes [21,22,23,24,25]. However, poor solubility in aqueous media and the instability of curcumin prevent its clinical application, so a newly synthesized curcumin analog, GO-Y078 ((1E,4E)-1-(4-hydroxy-3,5-dimethoxyphenyl)-5-(3,4,5-trimethoxyphenyl)-penta-1,4-dien-3-one) (Figure 1A), has been developed to enhance growth inhibition by directly interacting at the substrate-binding site and its solubility to overcome low bioavailability of curcumin [26,27,28]. In vivo GO-Y078 presents a 40% increase in survival time that is not obtained by curcumin in an experimental mouse model [26]. With increased bioavailability, GO-Y078 has yielded multi-target properties of new cancer chemotherapeutic effects over the past years [28,29,30]; nonetheless, the anti-cancer effect of GO-Y078 on osteosarcoma remains unclear. Hence, we investigated whether GO-Y078 affects cell apoptosis and the arrest of human osteosarcoma cells and attempted to define its underlying mechanisms.

## 2. Results

### 2.1. Cytotoxicity of GO-Y078 in Human Osteosarcoma U2OS, MG-63, 143B, and Saos-2 Cells

To assess the cytotoxicity of GO-Y078 on human osteosarcoma U2OS, MG-63, 143B, and Saos-2 cells, the MTT assay was utilized. After 24 h of treatment, the viabilities of U2OS, MG-63, 143B, and Saos-2 cells in the presence of concentrations of 1, 2, 4, 8, and 16 μM of GO-Y078 were significantly different from that of controls (0 μM) (Figure 1B) and all of the relationships were dose-dependent (*p* < 0.001, *p* < 0.001, *p* < 0.001, and *p* < 0.001, respectively). Moreover, a 24-h treatment with 8 μM of GO-Y078 showed a 41.5% reduction, while a 24-h treatment with 16 μM of GO-Y078 decreased 54.7% cell viability in U2OS cells. In MG-63 cells, those of 39.9% were reduced in 8 μM and 79.6% in 16 μM of GO-Y078. Similarly, reductions of 51.8% and 57.4% in 8 μM and 58.9% and 71.6% in 16 μM of GO-Y078 were observed in 143B and Saos-2 cells, respectively. Afterward, we used this concentration range of 1, 2, 4, and 8 μM for GO-Y078 in all subsequent experiments to explore its anti-cancer properties in U2OS and 143B cells.

### 2.2. GO-Y078 Induces Apoptosis and Sub-G1 Fraction Arrest of U2OS and 143B Cells

**Figure 2:**
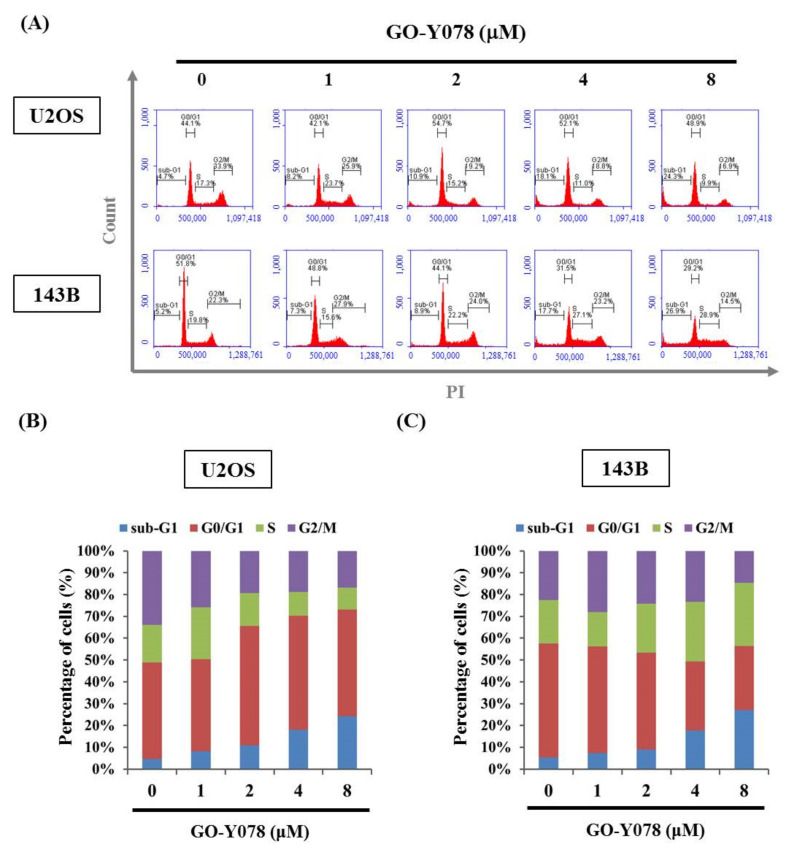
Effects of GO-Y078 on the cell cycle of U2OS and 143B cells. U2OS and 143B were treated with GO-Y078 (1, 2, 4, and 8 μM) for 24 h and then subjected to flow cytometry after (**A**) propidium iodine (PI) staining to analyze DNA contents. (**B**) The cell cycle profile of (**B**) U2OS and (**C**) 143B cells in flow cytometry was subsequently quantified.

To further examine the mechanism of GO-Y078*′*s inhibition of U2OS and 143B cell proliferation, the cell number percentage in each phase (G0/G1, S, and G2/M) of the cell cycle was calculated by flow cytometry. After 24 h of GO-Y078 (1, 2, 4, and 8 μM) treatment, the distribution of cells had markedly increased before the G1 phase (also called the sub-G1 fraction) from 4.7% to 24.3% and from 5.2% to 26.9% in U2OS and 143B, respectively (Figure 2A–C), suggesting that cell cycle arrest at the sub-G1 fraction may contribute to the cytotoxic effect.

**Figure 3:**
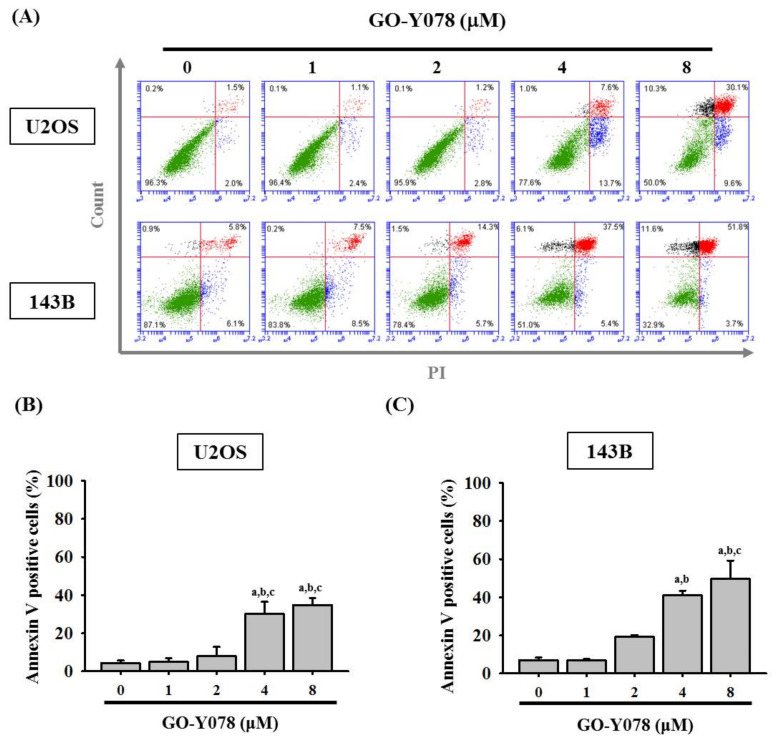
Effects of GO-Y078 on the cycle apoptosis in U2OS and 143B cells. U2OS and 143B were treated with GO-Y078 (1, 2, 4, and 8 μM) for 24 h and then subjected to flow cytometry after (**A**) PI and Annexin V-FITC/PI staining to analyze DNA contents. Cells that were considered viable were FITC Annexin V and PI negative; cells that were in early apoptosis were FITC Annexin V positive and PI negative; cells that were in late apoptosis or already dead were both FITC Annexin V and PI positive. (**B**) Thereupon quantitative analyses of early apoptosis and late apoptosis of (**B**) U2OS and (**C**) 143B cells were summed up to differentiate apoptosis from necrosis. Results are shown as mean ± S.D. *n* = 5. ANOVA analysis with Tukey’s posteriori comparison was used. U2OS: F = 47.309, *p* < 0.001; 143B: F = 9.663, *p* < 0.001. ^a^ Significantly different, *p* < 0.05, when compared to control. ^b^ Significantly different, *p* < 0.05, when compared to 1 μM. ^c^ Significantly different, *p* < 0.05, when compared to 2 μM.

To verify whether the suppressive effect of GO-Y078 on cell growth is derived from apoptosis rather than necrosis, an annexin V-FITC/PI apoptosis assay was performed to test the viability of U2OS and 143B cells before gross morphological changes because detection of apoptosis at early stages is critical for understanding the pathways of programmed cell death. After treating with up to 8 μM of GO-Y078 in U2OS and 143B cells for 24 h, flow cytometry showed apparent increases in both early (negative PI/positive annexin V-FITC) and late (positive PI/positive annexin V-FITC) apoptotic cells (Figure 3A). Connectedly, GO-Y078 significantly increased early and late (positive annexin V-FITC) apoptotic cells, corresponding to the sub-G1 fraction accumulation of U2OS and 143B cells (U2OS: *p* < 0.001 and 143B: *p* < 0.001) (Figure 3B,C).

### 2.3. GO-Y078 Increases Cleaved Caspase 3 and Decreases cIAP-1 and XIAP in U2OS and 143B Cells

**Figure 4:**
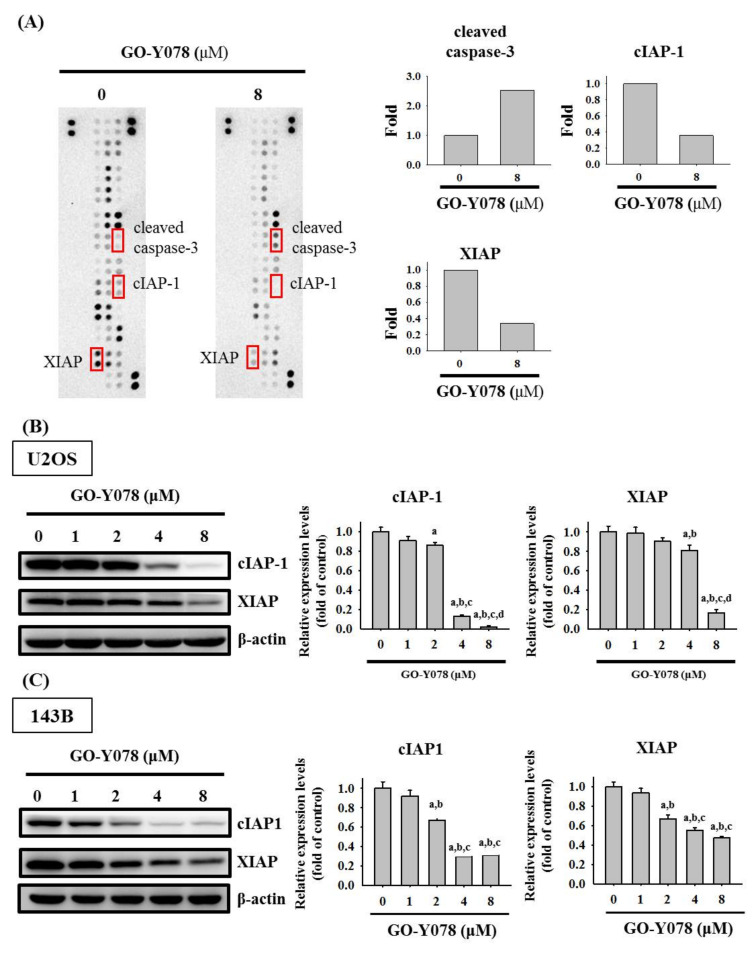
Effects of GO-Y078 on the human apoptosis array and IAPs in U2OS and 143B cells. (**A**) After treatment of 8 μM GO-Y078 for 24 h in U2OS cells, the human apoptosis array, 35 apoptosis-related proteins included, were employed as described in the Materials and Methods section and the increased protein cleaved caspase-3 and decreased proteins cIAP-1 and XIAP were subjected to quantitative analysis. Western blot analysis for cIAP-1 and XIAP after various concentrations (1, 2, 4, and 8 μM) of GO-Y078 treatment for 24 h in (**B**) U2OS and (**C**) 143B cells were measured as described in the Materials and Methods section. Subsequently, expressions of cIAP-1 and XIAP were subjected to quantitative analysis. Results are shown as mean ± S.D.; *n* = 3. ANOVA analysis with Tukey’s posteriori comparison was used. U2OS: cIAP-1: F = 611.988, *p* < 0.001; XIAP: F = 137.150, *p* < 0.001. 143B: cIAP-1: F = 178.003, *p* < 0.001; XIAP: F = 115.677, *p* < 0.001. ^a^ Significantly different, *p* < 0.05, when compared to control. ^b^ Significantly different, *p* < 0.05, when compared to 1 μM. ^c^ Significantly different, *p* < 0.05, when compared to 2 μM. ^d^ Significantly different, *p* < 0.05, when compared to 4 μM.

To identify the underlying mechanism of apoptosis induced by GO-Y078 in U2OS cells, we first employed the human apoptosis array to determine apoptosis-related proteins. Consequently, obvious increases of the cleaved caspase 3 protein and decreases of cIAP-1 and XIAP proteins in U2OS cells were observed after treatment of 8 μM GO-Y078 for 24 h, suggesting that the effector caspase 3, cIAP-1, and XIAP may actually be the responsible executioners of the U2OS cell (Figure 4A). To validate the observations in the human apoptosis array, the expression changes were detected by Western blotting and dose-dependent GO-Y078 decreases of cIAP-1 and XIAP expressions in U2OS and 143B cells were confirmed (U2OS: *p* < 0.001 and *p* < 0.001, respectively; 143B: *p* < 0.001 and *p* < 0.001, respectively) (Figure 4B,C).

**Figure 5:**
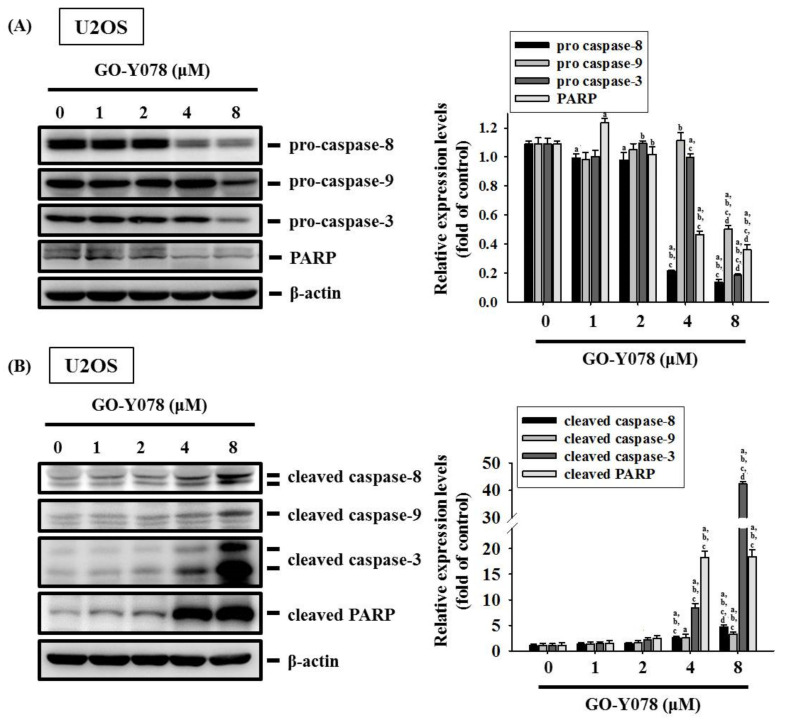

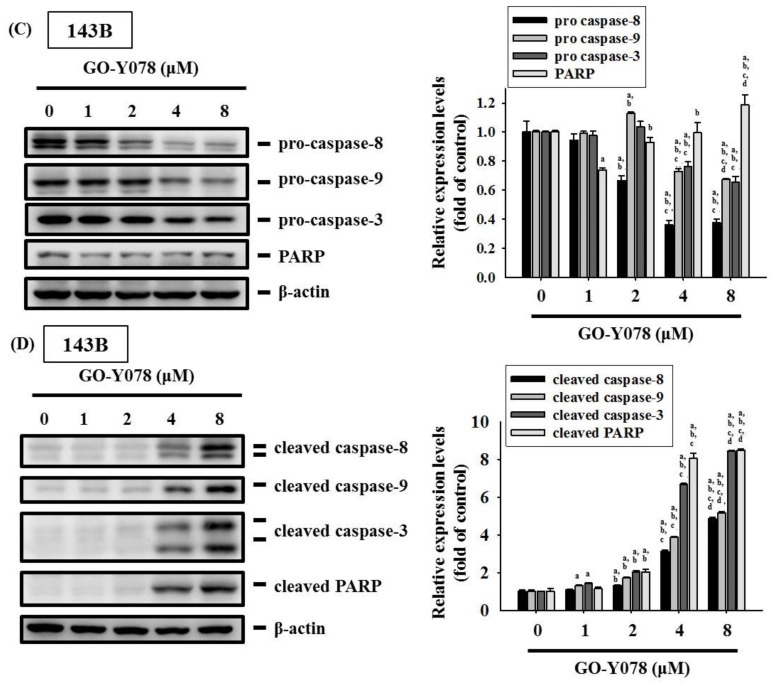
Effects of GO-Y078 on the activation of caspases 8, 9, and 3 in U2OS and 143B cells. Western blot analysis for (**A**) caspases 8, 9, and 3, and PARP as well as (**B**) their active forms after various concentrations (1, 2, 4, and 8 μM) of GO-Y078 treatment for 24 h in U2OS and (**C**,**D**) 143B cells were measured; subsequently, all of them were subjected to quantitative analysis. Results are shown as mean ± S.D.; *n* = 3. ANOVA analysis with Tukey’s posteriori comparison was used. U2OS: caspase 8: F = 677.981, *p* < 0.001; caspase 9: F = 98.434, *p* < 0.001; caspase 3: F = 485.708, *p* < 0.001; PARP: F = 414.042, *p* < 0.001; cleaved caspase 8: F = 83.446, *p* < 0.001; cleaved caspase 9: F = 11.128, *p* = 0.001; cleaved caspase 3: F = 2633.856, *p* < 0.001; cleaved PARP: F = 282.739, *p* < 0.001. 143B: caspase 8: F = 132.953, *p* < 0.001; caspase 9: F = 913.932, *p* < 0.001; caspase 3: F = 84.318, *p* < 0.001; PARP: F = 36.132, *p* < 0.001; cleaved caspase 8: F = 2408.883, *p* < 0.001; cleaved caspase 9: F = 3204.049, *p* < 0.001; cleaved caspase 3: F = 14889.882, *p* < 0.001; cleaved PARP: F = 1612.238, *p* < 0.001. ^a^ Significantly different, *p* < 0.05, when compared to control. ^b^ Significantly different, *p* < 0.05, when compared to 1 μM. ^c^ Significantly different, *p* < 0.05, when compared to 2 μM. ^d^ Significantly different, *p* < 0.05, when compared to 4 μM.

Moreover, the effector caspase 3 and its upstream initiators, caspases 8 and 9, as well as their cleaved forms were determined with Western blotting to investigate the effect of GO-Y078 on the caspase cascade in the apoptotic signaling pathway. After a treatment of different concentrations of GO-Y078 in U2OS and 143B cells for 24 h, the higher concentrations of GO-Y078 correspond to more expressions of the cleaved forms of caspases 8, 9, and 3, and poly adenosine diphosphate-ribose polymerase (PARP) dose-dependently (U2OS: *p* < 0.001, *p* = 0.001, *p* < 0.001 and *p* < 0.001, respectively; 143B: *p* < 0.001, *p* < 0.001, *p* < 0.001 and *p* < 0.001, respectively), combined with the lesser expressions of caspases 8, 9, and 3, and PARP dose-dependently (U2OS: *p* < 0.001, *p* < 0.001, *p* < 0.001 and *p* < 0.001, respectively; 143B: *p* < 0.001, *p* < 0.001, *p* < 0.001 and *p* < 0.001, respectively) (Figure 5A–D). In addition to the suppression of cIAP-1 and XIAP expressions, we found that GO-Y078 induces apoptosis in U2OS and 143B cells by activating both extrinsic caspase 8- and intrinsic caspase 9-mediated pathways and their downstream effector caspase 3 and PARP, enzymes involved in apoptosis.

### 2.4. GO-Y078 Activates Extrinsic and Intrinsic Apoptotic Processes via JNK and p38 Pathways in U2OS and 143B Cells

**Figure 6:**
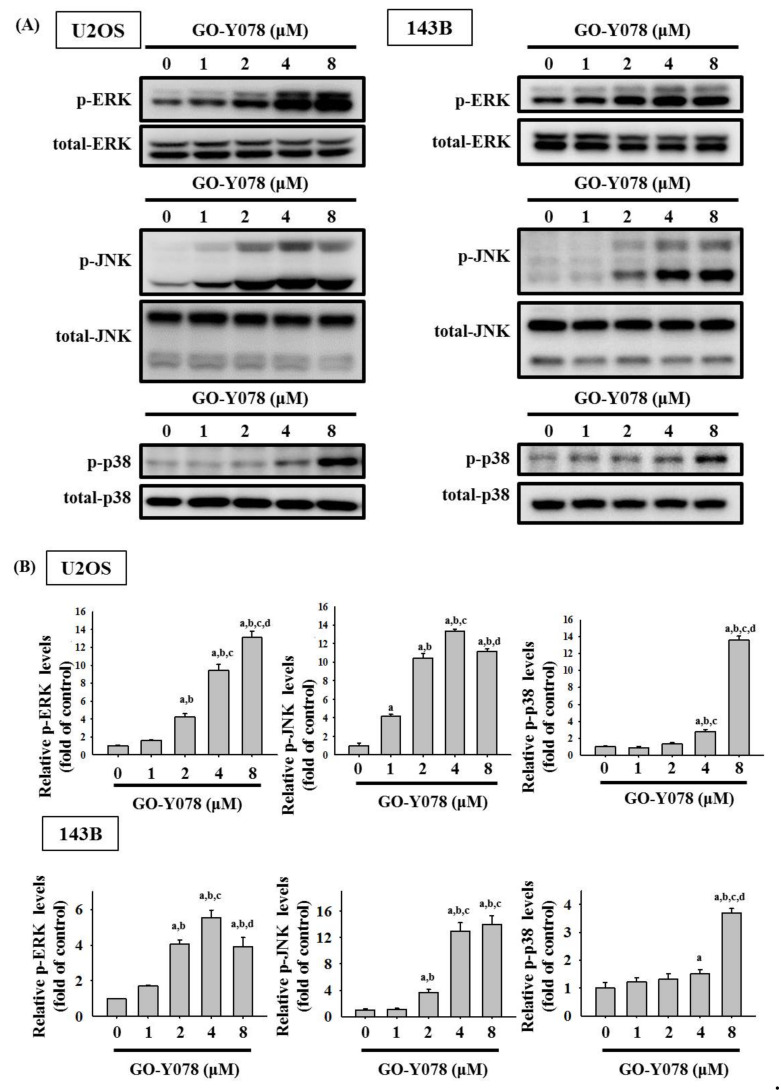
Effects of GO-Y078 on the phosphorylation of ERK, JNK, and p38 in U2OS and 143B cells. (**A**) Expressions of ERK1/2, JNK 1/2, and p38, as well as their phosphorylation after various concentrations (1, 2, 4, and 8 μM) of GO-Y078 treatment for 24 h in U2OS and 143B cells, were measured through Western blot analysis. (**B**) Next, they were subjected to quantitative analysis. Results are shown as mean ± S.D.; *n* = 3. ANOVA analysis with Tukey’s posteriori comparison was used. U2OS: p-ERK/ERK: F = 370.856, *p* < 0.001; p-JNK/JNK: F = 872.068, *p* < 0.001; p-p38/p38: F = 1181.680, *p* < 0.001. 143B: p-ERK/ERK: F = 2465.987, *p* < 0.001; p-JNK/JNK: F = 100.843, *p* < 0.001; p-p38/p38: F = 120.566, *p* < 0.001. ^a^ Significantly different, *p* < 0.05, when compared to control. ^b^ Significantly different, *p* < 0.05, when compared to 1 μM. ^c^ Significantly different, *p* < 0.05, when compared to 2 μM. ^d^ Significantly different, *p* < 0.05, when compared to 4 μM.

Since MAPK pathways have been implicated in playing an important role in the regulation of apoptosis and may be part of the signaling pathways that directly affect caspases 8, 9, and 3, and PARP, the Western blot analysis was employed to further investigate the underlying molecular mechanisms. As shown in Figure 6, GO-Y078 increased phosphorylation of ERK1/2, JNK1/2, and p38 dose-dependently in U2OS and 143B cells (U2OS: *p* < 0.001, *p* < 0.001 and *p* < 0.001, respectively; 143B: *p* < 0.001, *p* < 0.001 and *p* < 0.001), indicating that GO-Y078 activates phosphorylation of ERK1/2, JNK1/2, and p38 in U2OS and 143B cells.

**Figure 7:**
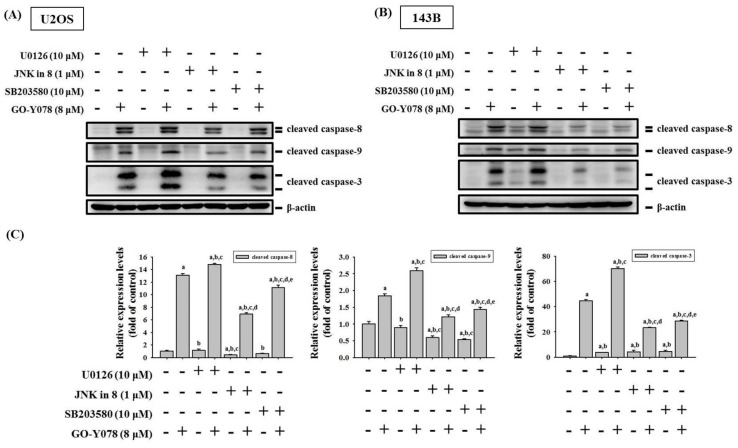

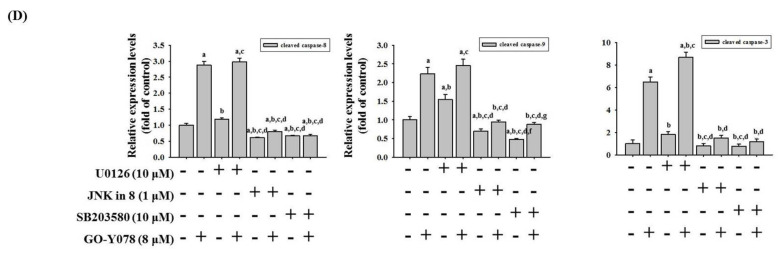
Effects of GO-Y078 and inhibitors of ERK1/2 (U0126), JNK1/2 (JNK-in-8), and p38 (SB203580) on cleaved caspases 8, 9, and 3 expressions of U2OS and 143B cells. Expressions of cleaved caspases 8, 9, and 3 after pretreatment with or without 10 μM of U0126, 1 μM of JNK-in-8, and 10 μM of SB203580 for 1 h followed by 8 μM or no GO-Y078 treatment for an additional 24 h in (**A**) U2OS and (**B**) 143B cells were measured through Western blot analysis. Next, they were subjected to quantitative analysis. Results are shown as mean ± S.D.; *n* = 3. −: without treatment; +: with treatment. ANOVA analysis with Tukey’s posteriori comparison was used. (**C**) U2OS: cleaved caspase 8: F = 2913.043, *p* < 0.001; cleaved caspase 9: F = 348.891, *p* < 0.001; cleaved caspase 3: F = 2804.587, *p* < 0.001. (**D**) 143B: cleaved caspase 8: F = 634.867, *p* < 0.001; cleaved caspase 9: F = 135.304, *p* < 0.001; cleaved caspase 3: F = 282.973, *p* < 0.001. ^a^ Significantly different, *p* < 0.05, when compared to control. ^b^ Significantly different, *p* < 0.05, when compared to 8 μM GO-Y078. ^c^ Significantly different, *p* < 0.05, when compared to U0126. ^d^ Significantly different, *p* < 0.05, when compared to JNK-in-8. ^e^ Significantly different, *p* < 0.05, when compared to SB203580.

To further identify whether activation of ERK1/2, JNK1/2, and p38 phosphorylation by GO-Y078 interferes with the actions of caspases 8, 9, and 3 of the extrinsic and intrinsic apoptotic processes in U2OS and 143B cells, we used inhibitors of ERK1/2 (U0126), JNK1/2 (JNK-in-8), and p38 (SB203580) with or without treatment of 8 μM GO-Y078 in Western blotting. As expected, cleaved caspases 8, 9, and 3 were activated by 8 μM of GO-Y078 in U2OS and 143B cells (U2OS: *p* < 0.05, *p* < 0.05 and *p* < 0.05, respectively; 143B: *p* < 0.05, *p* < 0.05 and *p* < 0.05, respectively) (Figure 7A–D). Intriguingly, inhibitors of JNK (JNK-in-8) and p38 (SB203580) significantly repressed GO-Y078′s increase in cleaved caspases 8, 9, and 3 in U2OS and 143B cells (U2OS: JNK-in-8: *p* < 0.05, *p* < 0.05 and *p* < 0.05, respectively; SB203580: *p* < 0.05, *p* < 0.05 and *p* < 0.05, respectively. 143B: JNK-in-8: *p* < 0.05, *p* < 0.05 and *p* < 0.05, respectively; SB203580: *p* < 0.05, *p* < 0.05 and *p* < 0.05, respectively), yet the inhibitor of ERK (U0126) did not suppress, but actually enhanced GO-Y078′s increase of cleaved caspases 3, 8, and 9 (U2OS: U0126: *p* < 0.05, *p* < 0.05 and *p* < 0.05, respectively; 143B: U0126: *p* < 0.05, *p* < 0.05 and *p* < 0.05, respectively). Altogether, these findings indicated that the JNK1/2 and p38 pathways play a critical upstream role in GO-Y078-mediated apoptosis of extrinsic caspase 8- and intrinsic caspase 9-mediated pathways and their downstream effector caspase 3 in U2OS and 143B cells.

## 3. Discussion

Curcumin is a well-known potential anti-cancer drug but with poor bioavailability due to its low aqueous solubility and the body’s rapid metabolism and systemic elimination. In the past decade, multifunctional micelles, liposomal nanoparticles, and liposomes followed by 3D printed scaffolds in a controlled delivery of curcumin to osteosarcoma cells were developed to accumulate targeting capacity for improving the therapeutic effect [31,32,33]. Moreover, several curcumin analogs have been also developed to improve poor bioavailability for chemotherapy in various cancers [27]. In human osteosarcoma, DK1 induces apoptosis in U2OS and MG-63 cells through the mitochondrial-dependent signaling pathway [34], and CH-5 triggers the apoptotic pathway in U2OS, MG-63, and Saos-2 cells via the modulation of transcription factors p53/Sp1 [35]. Also, we previously found that CLEFMA activates the extrinsic and intrinsic apoptotic processes through the JNK1/2 and p38 pathways in human osteosarcoma U2OS and HOS cells [36].

A synthesized curcumin analog, GO-Y078, developed by Shibata and Iwabuchi et al. exhibits at least 10 times higher growth-suppressive potential, 1.98 times higher solubility, 3.8 times higher the increase in the subG1 fraction, and 3.47 times higher inducer of apoptosis than curcumin and it induces apoptosis through numerous mechanisms just as curcumin [26]. The concentration of the GO-Y078 compound in ethanol-cremophor EL^®^ reached 300 mM and no aggregations were seen, whereas curcumin and its analogs are usually dissolved in dimethyl sulfoxide (DMSO) as a stock solution due to nontoxic at 1% DMSO alone [26,30]. Furthermore, GO-Y078 has been reported to bear additional characteristics of improved solubility and effectiveness in a mouse model [26], but no research of GO-Y078 has been reported on the apoptotic program in human osteosarcoma cells. In this study, we intriguingly demonstrated GO-Y078′s cytotoxicity on U2OS, MG-63, 143B, and Saos-2 cells and used a flow cytometry assay for cell cycle and apoptosis to confirm that GO-Y078 could interfere with the cell cycle at the sub-G1 fraction and induce apoptosis in U2OS and 143B cells.

Apoptosis is triggered by the extrinsic and intrinsic apoptotic processes, and activation of the execution of the apoptotic program is determined by a delicate balance between pro-apoptotic proteins, such as caspases 8, 9, and 3, and anti-apoptotic proteins [9,36]. As members of the anti-apoptotic family of proteins, IAP molecules, such as cIAP-1 and XIAP, play important roles in modulating the inactivation of apoptosis and correlate with a poor prognosis and resistance to chemotherapy in osteosarcoma [14,15,16]. Therefore, targeting IAPs is an attractive therapeutic method undertaken for the development of a fascinating strategy of anti-osteosarcoma therapies [37], and some IAP inhibitors are currently used in clinical trials as monotherapy or combination in chemotherapy to improve the therapeutic effect. As there is an increased expression of XIAP in osteosarcoma, silencing XIAP represses cell growth and promotes the sensitivity of osteosarcoma cells to doxorubicin and cisplatin [16]. Herein, we explored dual targeting by stimulation of the extrinsic and intrinsic apoptotic pathways and suppression of cIAP-1 and XIAP in GO-Y078-treated U2OS and 143B cells. Through collecting information from various aspects of signal transduction cascades and IAPs, both pathways continuously process this signaling and eventually decide on the fate of U2OS and 143B cells.

While GO-Y078′s phosphorylation of ERK1/2, JNK1/2, and p38 in U2OS and 143B cells was observed in the study, we supposed that GO-Y078′s induction of the extrinsic and intrinsic apoptotic pathways was through these three MAPK pathways. However, the GO-Y078′s increases in cleaved caspases 3, 8, and 9 could be expectedly inhibited by co-treatment with inhibitors of JNK (JNK-in-8) and p38 (SB203580), but co-treatment with the ERK inhibitor (U0126) had no effect on the increased effect. Therefore, these findings suggested that GO-Y078 activates both extrinsic and intrinsic apoptotic pathways in U2OS and 143B cells through JNK and p38 signaling, but the ERK pathway is not involved. In addition to the suppression of IAPs, we demonstrated that GO-Y078 executes both extrinsic and intrinsic apoptotic signals in U2OS and 143B cells via the activation of the JNK and p38 pathways.

## 4. Materials and Methods

### 4.1. Materials

Cell culture materials including Dulbecco’s modified Eagle medium (DMEM) and fetal bovine serum (FBS) were purchased from Gibco-BRL (Gaithersburg, MD, USA) and Hyclone Laboratories, Inc. (Logan, UT, USA), respectively. Antibodies specifically for p38, phosphorylated p38, caspase 3, propidium iodine (PI), and FITC (fluorescein isothiocyanate-labeled) Annexin V Apoptosis Detection Kit I were obtained from BD Biosciences (San Jose, CA, USA). The Human Apoptosis Array Kit was purchased from R&D Systems (Minneapolis, MN, USA). Additionally, antibodies specifically for ERK1/2, JNK1/2, phosphorylated ERK1/2 and JNK1/2, caspases 8 and 9, and cleaved caspases 3, 8, and 9 were purchased from Cell Signaling Technology (Danvers, MA, USA). Unless otherwise specified, all chemicals used in this study, including streptomycin, were purchased from Sigma-Aldrich (St. Louis, MO, USA).

### 4.2. Cell Culture and GO-Y078 Treatment

Being purchased from the Food Industry Research and Development Institute (Hsinchu, Taiwan), the human osteosarcoma U2OS (15-year-old female), MG-63 (14-year-old male), and 143B (11-year-old Caucasian female) cells were supplemented with 10% FBS, 1% penicillin/streptomycin, and 5 mL glutamine, and cultured in DMEM. Saos-2 (osteogenic sarcoma; 11-year-old Caucasian female) cells, obtained from American Type Culture Collection (Manassas, VA, USA), were cultured in DMEM supplemented with 10% FBS and 1% penicillin (100 U/mL)/streptomycin (100 μg/mL). The cell cultures were maintained at 37 °C in a humidified atmosphere of a 5% CO_2_ incubator. Further, 100% DMSO and GO-Y078 were purchased from Merk, Darmstadt, Germany (Darmstadt, Germany) and Tokyo Chemical Industry Co., Ltd. (Tokyo, Japan), respectively. GO-Y078 was dissolved initially in 100% DMSO to achieve an 8 mM stock solution of GO-Y078, and appropriate amounts of stock solution were subsequently added into the culture medium to achieve the indicated concentrations. While the final concentration of DMSO in the medium was 0.1% (*v/v*) [26], which has shown to be nontoxic in 4 osteosarcoma cell lines, 0.1% DMSO solution (solvent) without GO-Y078 was used as the blank reagent [9,36,38,39,40,41,42].

### 4.3. Microculture Tetrazolium (MTT) Assay

To obtain information regarding the effect of apoptosis induced by GO-Y078, we subjected 8.0 × 10^4^/mL U2OS cells, 7.0 × 10^4^/mL MG-63 cells, 2.0 × 10^5^/mL 143B cells, and 1.0 × 10^5^/mL Saos-2 cells in 24-well plates for 16 h and treated them with different concentrations (1, 2, 4, 8, and 16 μM) of GO-Y078 to assay the cell viability via MTT (3-(4,5-dimethylthiazol-2-yl)-2,5-diphenyltetrazolium bromide) assay as described previously [38,39].

### 4.4. Flow Cytometric Analysis

To estimate the proportion of U2OS and 143B cells in different phases of the cell cycle affected by GO-Y078, cellular DNA contents and apoptosis were measured via flow cytometry as stated previously [40]. Briefly, we cultured 8.0 × 10^5^ U2OS cells and 1.2 × 10^5^ 143B cells in 6-cm dishes and treated them with different concentrations (0, 1, 2, 4, and 8 μM) of GO-Y078 for 24 h. After staining with PI, 2.0 × 10^5^ U2OS cells and 143B cells in one Eppendorf tube, the cell cycle was analyzed on a BD Accuri^TM^ C6 Plus personal flow cytometer (BD Biosciences, San Jose, CA, USA).

### 4.5. Annexin V-FITC Apoptosis Staining Assay

Membrane phospholipid phosphatidylserine molecules could be translocated from the inner face of the plasma membrane to the cell surface soon after initiating apoptosis. Annexin V, a fluorescent conjugated protein with a high affinity for phospholipid phosphatidylserine, was used as a stain for identifying apoptosis at an earlier stage than PI staining for DNA fragmentation. We cultured 8.0 × 10^5^ U2OS cells and 1.2 × 10^5^ 143B cells in one 6-cm dish and treated them with different concentrations (0, 1, 2, 4, and 8 μM) of GO-Y078 for 24 h. Subsequently, U2OS and 143B cells were harvested with trypsinization together with floating non-viable cells. The FITC Annexin V Apoptosis Detection Kit I was performed according to the manufacturer’s protocols (BD Biosciences, San Jose, CA, USA); thereafter, the cell cycle analysis was measured by flow cytometry. Combined with PI staining, annexin V-FITC apoptosis staining was performed to differentiate apoptosis from necrosis as reported previously [36].

### 4.6. Human Apoptosis Array

To explore the underlying mechanism of induced apoptosis, a Human Apoptosis Array Kit was used to evaluate protein lysates of 2.0 × 10^6^ U2OS cells/dish and 1.2 × 10^5^ 143B cells/dish from vehicle- or 8 μM GO-Y078-treated for 24 h according to the manufacturer’s protocols (R&D Systems, Minneapolis, MN, USA). The kit detected a total of 35 human apoptosis-related proteins simultaneously. Captured proteins were presented on the nitrocellulose membrane, detected with biotinylated detection antibodies, then finally visualized using chemiluminescent detection reagents.

### 4.7. Protein Extraction and Western Blot Analysis

To investigate the molecular mechanism further, the initiator and effector caspases and signaling pathways were detected using Western blot analysis. As described previously, 8.0 × 10^5^/dish U2OS cells and 1.2 × 10^5^/dish 143B cells were cultured in 6 cm plates for 16 h and treated with different concentrations (0, 1, 2, 4, and 8 μM) of GO-Y078 for 24 h, and the total cell lysates of U2OS and 143B cells were prepared [38,39,41]. We performed Western blot analysis with specific primary antibodies against caspases 3, 8, and 9, PARP, cIAP-1, XIAP, cleaved caspases 3, 8, and 9, and the specific antibodies for unphosphorylated or phosphorylated forms of the three corresponding MAPKs (ERK1/2, JNK1/2, and p38). Blots were then incubated with a horseradish peroxidase goat anti-rabbit or anti-mouse IgG for 1 h and the intensity of each band was measured via densitometry as depicted previously [38,39,41].

### 4.8. Statistical Analysis

Statistical calculations of the data were performed using one-way analysis of variance with post hoc Scheffe’s and Tukey’s tests for more than two groups with unequal and equal sample sizes per group, respectively. Each experiment was performed in triplicate at least (*n* ≥ 3) were performed. Statistical significance was at *p* < 0.05.

## 5. Conclusions

Overall, these results revealed that GO-Y078 decreases cell viabilities of human osteosarcoma U2OS, MG-63, 143B, and Saos-2 cells and induces cell arrest in the sub-G1 fraction and apoptosis of U2OS and 143B cells. Through dual effects of repressing IAPs and activating JNK and p38 pathways, both the extrinsic and intrinsic caspase cascades are triggered to induce apoptosis and sub-G1 fraction cell arrest of U2OS and 143B cells. Thus, GO-Y078 may be a potential strategy for treating human osteosarcoma, whereas further tests to investigate the therapeutic potential of GO-Y078 combined with chemotherapy in osteosarcoma treatment is needed in the future.

## Data Availability

The data presented in this study are available on request from the corresponding author.
